# A conserved allosteric element controls specificity and activity of functionally divergent PP2C phosphatases from *Bacillus subtilis*

**DOI:** 10.1016/j.jbc.2021.100518

**Published:** 2021-03-06

**Authors:** Kristin Ho, Niels Bradshaw

**Affiliations:** Department of Biochemistry, Brandeis University, Waltham, Massachusetts, USA

**Keywords:** allosteric regulation, *Bacillus*, protein phosphatase 2C (PP2C), stress response, substrate specificity, bacterial transcription, bacterial protein phosphatase, bacterial signal transduction, cell signaling, metal ion–protein interaction, LB, lysogeny broth, PP2C, protein phosphatase 2C, X-gal, 5-bromo-4-chloro-3-indolyl-β-D-galactopyranoside

## Abstract

Reversible phosphorylation relies on highly regulated kinases and phosphatases that target specific substrates to control diverse cellular processes. Here, we address how protein phosphatase activity is directed to the correct substrates under the correct conditions. The serine/threonine phosphatase SpoIIE from *Bacillus subtilis*, a member of the widespread protein phosphatase 2C (PP2C) family of phosphatases, is activated by movement of a conserved α-helical element in the phosphatase domain to create the binding site for the metal cofactor. We hypothesized that this conformational switch could provide a general mechanism for control of diverse members of the PP2C family of phosphatases. The *B. subtilis* phosphatase RsbU responds to different signals, acts on a different substrates, and produces a more graded response than SpoIIE. Using an unbiased genetic screen, we isolated mutants in the α-helical switch region of RsbU that are constitutively active, indicating conservation of the switch mechanism. Using phosphatase activity assays with phosphoprotein substrates, we found that both phosphatases integrate substrate recognition with activating signals to control metal-cofactor binding and substrate dephosphorylation. This integrated control provides a mechanism for PP2C family of phosphatases to produce specific responses by acting on the correct substrates, under the appropriate conditions.

Protein kinases and phosphatases regulate diverse biological processes by controlling the phosphorylation state of target proteins, requiring that they select specific substrates and act on them at the correct time and place. Protein kinases have conserved allosteric elements that control kinase activity in response to regulatory inputs and have deep active site grooves that facilitate recognition of cognate substrates (1, 2, 3). Although all protein kinases belong to a single superfamily of related enzymes, protein phosphatases come from multiple evolutionarily unrelated lineages (4). Determining how each family of phosphatases achieves regulation and specificity will reveal mechanistic differences that distinguish each phosphatase family and may suggest specific strategies for targeting phosphatases with small molecules.

We focus here on the protein phosphatase 2C (PP2C) family of serine/threonine phosphatases, members of which are found across all kingdoms of life (5, 6). Phosphatases from the PP2C family are precisely regulated to control a diverse range of processes including stress response, development, virulence, and cell growth and death in all kingdoms of life, but their mechanisms of regulation and substrate specificity are unknown (4, 7). To address this question, we developed a system to compare substrate selection and regulation of two PP2C phosphatases, SpoIIE and RsbU, which coexist in the same bacterial cell but respond to different signals and act on distinct phosphoprotein substrates.

SpoIIE controls the developmental program of spore development in the bacterium *Bacillus subtilis* (Fig. 1*A*) (8). After asymmetric cell division, SpoIIE is stabilized against proteolysis and is activated by multimerization in small cells (9). The multimerization, stabilization, and activation of SpoIIE are mediated by its unique regulatory domain. Upon activation, SpoIIE dephosphorylates SpoIIAA to activate a cell-specific transcription factor (Fig. 1*A*) (8, 10). We recently found that SpoIIE is activated by rotation of an α-helical element at the base of the phosphatase domain to form the binding site for the catalytically essential metal ions (we refer to the metal ions as “metal cofactor” and use Mn^2+^ as the metal for all experiments in this study, Fig. 1*B* and *C*) (11). The regulatory α-helical element we described is a conserved feature of the PP2C domain, and we hypothesized that it is preserved as a regulatory switch for phosphatases in this family (11).

**Figure 1:**
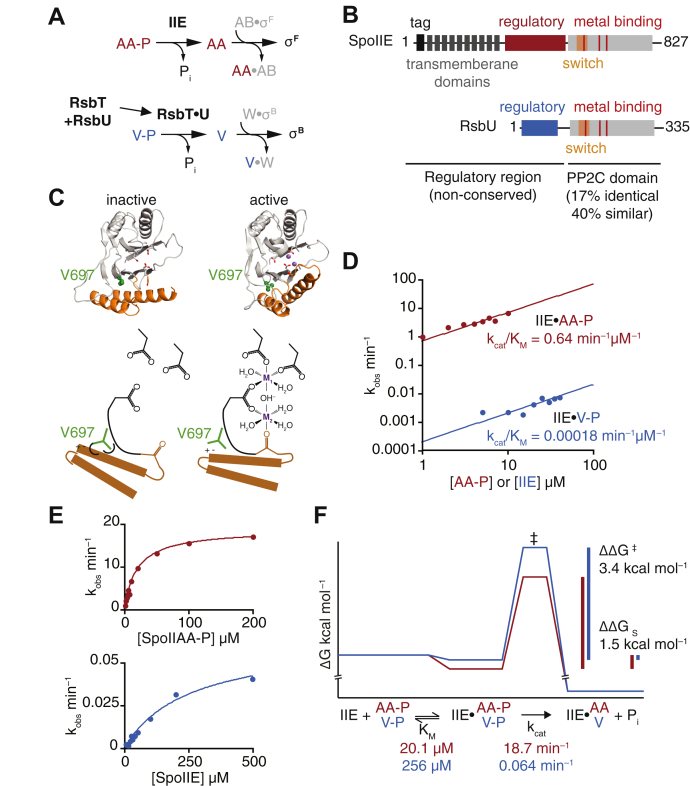
**SpoIIE discriminates between cognate and noncognate substrates.***A*, the pathways controlling the activity of the transcription factors σ^F^ and σ^B^ are diagrammed. The phosphatases SpoIIE (IIE) and RsbU dephosphorylate paralogous substrate proteins SpoIIAA-P (AA-P) and RsbV-P (V-P). The activity of RsbU is dependent upon activation by RsbT. In the unphosphorylated state, SpoIIAA and RsbV bind to anti-sigma factor proteins SpoIIAB (AB) and RsbW (W), displacing and activating the sigma factors. *B*, domain diagrams of SpoIIE and RsbU. The N-terminal regulatory regions of these proteins are unique, while the PP2C domains share 17% identity. The regulatory region of SpoIIE contains an N-terminal degradation tag (*black*) followed by 10 transmembrane domains (*gray*) (aa 49–320). Both SpoIIE (*magenta*) (aa 320–589) and RsbU (*blue*) (aa) contain unrelated regulatory domains. The PP2C domain of SpoIIE (aa 590–827) has been crystallized (11) and contains an α-helical regulatory switch (*orange*), which coordinates the binding of metal cofactor along with metal binding residues D795, D746, and D628 (*red*) through positioning the backbone carbonyl of G629. The PP2C domain of RsbU (aa 121–335) has not been crystallized but is predicted to contain a similar α-helical regulatory switch. The metal binding residues show conservation between the two proteins (the detailed alignment can be seen in Fig. S1). *C*, structures of SpoIIE in the inactive (*top left*, PDB ID: 5MQH) and active (*top right*, PDB ID: 5UCG) states. The α-helical switch is represented in *orange* and the side chains that coordinate divalent cations in the active site are shown as *sticks*. Manganese ions are modeled in the active site of SpoIIE from the structure of metal-bound RsbX and are represented as *spheres*. Valine 697 is shown as *green spheres*. Below each structure is a cartoon representation of the active site as it is controlled by the switch. In the inactive state, residue V697, shown in *green*, is modeled to be in a hydrophobic pocket, while in the active site, it is exposed to a hydrophilic patch. *D*, SpoIIE^590–827^ phosphatase activity with SpoIIAA-P (*red*) or RsbV-P (*blue*) as the substrate. Reactions with SpoIIAA-P as the substrate were performed as multiple turnover reactions with 0.1-μM SpoIIE^590–827^ and varying concentrations of SpoIIAA-P. Reactions with RsbV-P were performed as single turnover reactions (due to the extremely slow rates of reaction), using trace concentrations of RsbV-P with varying concentrations of SpoIIE^590–827^. All reactions contained 10 mM MnCl_2_. Data are fit to the linear equation observed rate = (*k*_cat_/K_M_)∗[SpoIIAA-P or SpoIIE] and are plotted on log/log axes. *E*, plots of the data from panel *C* including higher concentrations of SpoIIAA-P and SpoIIE. Data are fit using nonlinear curve fitting (KaleidaGraph) to the equation observed rate = *k*_cat_∗[SpoIIAA-P or SpoIIE]/(K_M_ + [SpoIIAA-P or SpoIIE]). *F*, a reaction coordinate diagram summarizing the data from panels *D* and *E*. PP2C, protein phosphatase 2C.

The phosphatase RsbU has a PP2C domain that is related to SpoIIE (17% sequence identity and 40% similarity and includes a region predicted to form the α-helical switch) (Fig. S1, *A* and *B*) but has an unrelated N-terminal regulatory domain (Fig. 1*B* and Fig. S1*A*) and responds to different signals to initiate the general stress response in *B. subtilis* (Fig. 1*A*) (12). The general stress response describes a broad transcriptional response that is activated by diverse bacterial species in response to changing conditions and coordinates processes including virulence, antibiotic resistance, and biofilm formation (13). A common mechanism for activation of a general stress response relies on a PP2C phosphatase that recognizes the initiating signal. This appears to be the most evolutionarily ancient and broadly conserved function of the PP2C family of phosphatases across the bacterial kingdom. To activate the general stress response under conditions of environmental stress in *B. subtilis*, RsbU binds to an activating protein (RsbT) and dephosphorylates RsbV. RsbV then activates the transcription factor σ^B^, which controls the general stress response transcriptional program in *B. subtilis* (Fig. 1*A*) (12). It is essential that SpoIIE and RsbU act with high fidelity on their cognate substrates (14); aberrant dephosphorylation of RsbV-P and activation of the general stress response during sporulation blocks spore development (15), and dephosphorylation of SpoIIAA-P in nonsporulating cells is toxic.

In addition to sensing different signals and acting on different substrates, SpoIIE and RsbU exhibit different response profiles. Whereas SpoIIE initiates a switch-like developmental transition, RsbU initiates a graded response with amplitudes scaled to the strength of the initiating signal (16, 17). These phosphatase–substrate pairs thus provide an experimentally tractable system within a well-characterized model organism with which to identify principles for how PP2C family of phosphatases are regulated and achieve substrate specificity.

Using a combined forward-genetic and biochemical approach to probe the regulation and specificity of RsbU and SpoIIE, we found that the regulatory switch is a conserved element that ensures each phosphatase is only active at the correct time, under the correct conditions, and when in complex with the correct substrate protein.

## Results

### SpoIIE principally achieves specificity by stabilizing the transition state

To assess how SpoIIE achieves specificity for its cognate substrate SpoIIAA as opposed to its noncognate substrate RsbV, we used the C-terminal phosphatase domain in isolation (SpoIIE^590–827^) (Fig. 1*C*). We selected this construct to test the hypothesis that specificity can be encoded by the PP2C catalytic domain alone and to identify potentially conserved mechanisms of specificity. At least three steps of the phosphatase reaction could in principle contribute to specificity: substrate recognition, chemistry, and metal-cofactor binding. To isolate the contributions of substrate recognition and chemistry, we held the metal-cofactor constant at saturating levels (10 mM MnCl_2_). Under these conditions, SpoIIE^590–827^ was specific, dephosphorylating SpoIIAA-P approximately 3500-fold more efficiently than the off-pathway substrate, RsbV-P (SpoIIAA-P *k*_cat_/K_M_ = 0.68 ± 0.04 min^−1^ μM^−1^, RsbV-P *k*_cat_/K_M_ = 0.00020 ± 0.00002 min^−1^ μM^−1^) (Fig. 1*D*). This specificity was principally the result of an increased *k*_cat_ for the cognate substrate (18.7 ± 0.4 min^−1^ for SpoIIAA, 0.064 ± 0.005 min^−1^ for RsbV) but was enhanced by a lower K_M_ for the cognate substrate (20 ± 1 μM for SpoIIAA, 255 ± 39 μM for RsbV) (Fig. 1*E*). Thus, specificity is achieved by directing 3.4 kcal/mol of the specific binding energy for the cognate substrate to stabilizing the transition state and 1.5 kcal/mol to stabilization of the enzyme substrate complex (Fig. 1*F*). Here we discuss K_M_ as representing “substrate recognition”, but also note that the lack of a lag phase in presteady state reactions, the μM K_M_ values for substrate, and minute timescale *k*_cat_ are all suggestive of the K_M_ being equal to the K_D_ of the enzyme–substrate complex.

### The regulatory switch couples substrate recognition to metal-cofactor binding

**Figure 2:**
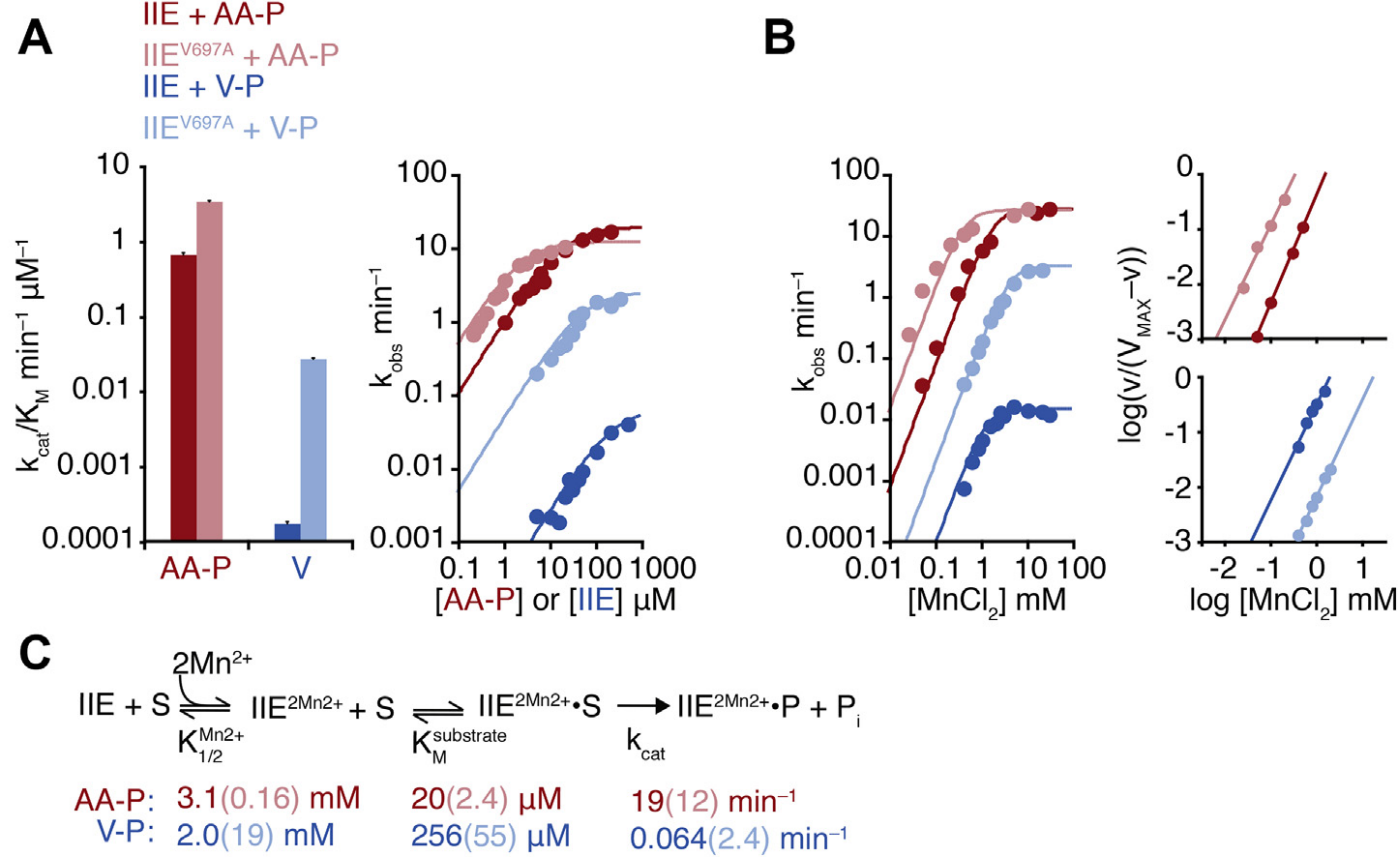
**SpoIIE specificity is controlled by the switch.***A*, plots showing the dephosphorylation of SpoIIAA-P (*red*) or RsbV-P (*blue*). The bar plot (*left*) summarizes the impact of the V697A substitution on the *k*_cat_/K_M_ of SpoIIE^590–827^. Data from SpoIIE^V697A^ are displayed in *light colors*. Reactions with SpoIIAA-P as the substrate were performed as multiple-turnover reactions with varying concentrations of SpoIIAA-P and 0.04 μM SpoIIE^590–827 V697A^ (the reduced K_M_ for this variant and SpoIIAA-P necessitated a lower concentration than used for SpoIIE^590–827^). Slow reactions with RsbV-P were performed as single-turnover reactions (necessary due to slow rates of reaction), using trace concentrations of RsbV-P with varying concentrations of SpoIIE^590–827 V697A^. Data for wt SpoIIE are from the same experiment as shown in Figure 1*D*. All reactions contained 10 mM MnCl_2_. To calculate *k*_cat_/K_M_, data from concentrations below the K_M_ are fit to the linear equation observed rate = (*k*_cat_/K_M_)∗[SpoIIAA-P or SpoIIE]. Error bars are the error of the fit. *Right* is a plot of the data but including higher concentrations of SpoIIAA-P and SpoIIE. Data are fit using nonlinear curve fitting (KaleidaGraph) to the equation observed rate = *k*_cat_∗[SpoIIAA-P or SpoIIE]/(K_M_ + [SpoIIAA-P or SpoIIE]). *B*, SpoIIE activity as a function of MnCl_2_ concentration, with SpoIIAA-P (*red*) or RsbV-P (*blue*). Multiple turnover reactions with SpoIIAA-P as the substrate included 0.1 μM SpoIIE and 50 μM SpoIIAA-P. Single turnover reactions with RsbV-P as the substrate included 50 μM SpoIIE and trace RsbV-P. Data are fit to a cooperative model using the equation k_obs_ = *k*_cat_∗[MnCl_2_]^2^/(K_1/2_ + [MnCl_2_]^2^). Hill plots from the data are shown to the *right* and are fit to a linear equation. For SpoIIE^590–827^ with SpoIIAA-P, *h* is 2.0 (1.7 for V697A) and 1.7 with RsbV-P (1.8 for V697A). *C*, a summary of kinetic parameters in the reaction scheme for dephosphorylation by SpoIIE. S indicates the phosphoprotein substrate, and P indicates the product.

The conserved regulatory switch moves to activate SpoIIE by recruiting a metal cofactor to the active site (Fig. 1*C*). We hypothesized that substrate binding is favored when the switch is in the active conformation, coupling substrate binding to metal-cofactor binding. To investigate this hypothesis, we took advantage of a gain-of-function mutant of SpoIIE (valine 697 to alanine, V697A) in which substitution of a single amino acid in the hydrophobic core of the phosphatase domain biases the switch to the active state (Fig. 1*C*) (11, 18, 19). The V697A substitution decreased the specificity of SpoIIE, increasing the *k*_cat_/K_M_ for RsbV-P 150-fold compared with a 5-fold increase for SpoIIAA-P (Fig. 2*A*). In the experiments described below, we analyzed the effect of the V697A substitution on K_M_, *k*_cat_, and metal-cofactor binding with both the cognate and noncognate substrates.

#### Substrate recognition (K_M_)

The V697A substitution decreased the K_M_ of SpoIIE^590–827^ for both SpoIIAA-P (10-fold) and RsbV-P (5-fold) (Fig. 2*A*), suggesting that substrate binding is favored when SpoIIE is in the active conformation.

#### Chemistry (*k*_cat_)

The V697A substitution increased the maximal catalytic rate (*k*_cat_) of SpoIIE^590–827^ for the noncognate substrate nearly 40-fold while having a modest effect on the cognate substrate (Fig. 2*A*). Thus, the switch controls the maximum catalytic rate of SpoIIE in addition to controlling metal-cofactor binding. Furthermore, the conformational flexibility of the switch can differentially affect the catalytic rate for cognate and noncognate substrates.

#### Metal-cofactor binding

To monitor metal-cofactor recruitment, we held the substrate concentration constant and measured SpoIIE activity with varied concentrations of MnCl_2_ (Fig. 2*B*). Consistent with our previous observations, the V697A substitution decreased the concentration of MnCl_2_ required for SpoIIE^590–827^ to dephosphorylate SpoIIAA-P (K_1/2_ 3.1 ± 0.9 mM for WT SpoIIE^590–827^ and K_1/2_ 0.16 ± 0.05 mM for SpoIIE^590–827^ V697A). In contrast, the V697A substitution increases the MnCl_2_ concentration required for dephosphorylation for the noncognate substrate RsbV-P (K_1/2_ of 2.0 ± 0.4 mM for WT SpoIIE^590–827^ and K_1/2_ of 19 ± 2 mM for SpoIIE^590–827^ V697A). Cooperativity of metal-cofactor binding (Hill coefficient of two, inset panels Fig. 2*B*) yields a switch-like response profile, appropriate for initiation of a developmental transition.

Together, these kinetic parameters demonstrate that the switch mediates coupling between metal-cofactor binding, substrate recognition, and chemistry and that the V697A substitution shifts binding energy for the noncognate substrate from metal-cofactor binding to chemistry, reducing specificity at saturating metal-cofactor concentrations (summarized in Fig. 2*C*).

### The SpoIIE regulatory domain controls metal-cofactor binding, substrate recognition, and chemistry

**Figure 3:**
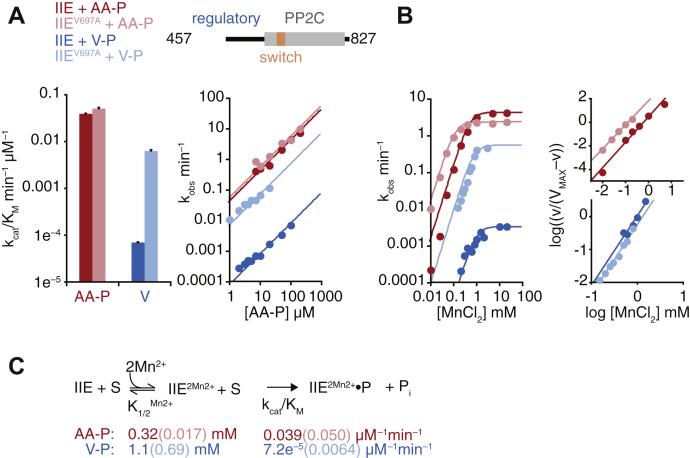
**SpoIIE activity and specificity are influenced by the regulatory domain.***A*, plots showing the dephosphorylation of SpoIIAA-P (*red*) and RsbV-P by a fragment of SpoIIE (SpoIIE^457–827^) that includes part of the regulatory domain. The fragment of the regulatory domain is diagramed above in *blue* with the phosphatase domain in *gray* and the switch in *orange*. Data from SpoIIE^457–827 V697A^ are displayed in *light colors*. The *k*_cat_/K_M_ values displayed in the *bar plot* are derived from the data displayed in the *right graph* with concentrations below the K_M_ fit to the linear equation observed rate = (*k*_cat_/K_M_)∗[SpoIIAA-P or SpoIIE]. Error bars are the error of the fit. Data in the right plot were fit using nonlinear curve fitting (KaleidaGraph) to the equation observed rate = *k*_cat_∗[SpoIIAA-P or SpoIIE]/(K_M_^MnCl2^ + [SpoIIAA-P or SpoIIE]). Reactions with SpoIIAA-P as the substrate were performed as multiple-turnover reactions with 0.1 μM SpoIIE, 10 mM MnCl_2_, and varying concentration of SpoIIAA-P. Reactions with RsbV-P as the substrate were performed as single-turnover reactions (necessary due to slow rates of reaction) with 10 mM MnCl_2_, trace RsbV-P, and varying concentrations of SpoIIE. *B*, SpoIIE^457–827^ activity as a function of MnCl_2_ concentration, with SpoIIAA-P (*red*) or RsbV-P (*blue*). Data are fit to a cooperative model using the equation k_obs_ = *k*_cat_∗[MnCl_2_]^2^/(K_1/2_ + [MnCl_2_]^2^). Hill plots from the data are shown to the *right* and are fit to a linear equation. Data for SpoIIE^457–827^ and SpoIIE^457–827•V697A^ with SpoIIAA-P as the substrate are from the study by Bradshaw *et al.* 2017, replotted here for reference. Reactions with RsbV-P as the substrate included 50-μM SpoIIE and trace RsbV-P. *C*, a summary of kinetic parameters in the reaction scheme for dephosphorylation by SpoIIE. S indicates the phosphoprotein substrate, and P indicates the product.

PP2C family of phosphatases has regulatory domains that control their activity (4, 11). We therefore repeated the above analysis with the phosphatase domain of SpoIIE together with a portion of the regulatory domain (SpoIIE^457–827^). We chose this fragment because it is a well-behaved soluble protein whose crystal structure we previously reported (11) (Fig. 3*A*). Although the regulatory domain is truncated, this fragment allowed us to test how contact with an accessory domain affects the behavior of the switch. Similar to our observations with SpoIIE^590–827^, SpoIIE^457–827^ was specific for SpoIIAA-P (SpoIIAA-P *k*_cat_/K_M_ = 0.039 ± 0.002 min^−1^ μM^−1^, RsbV-P *k*_cat_/K_M_ = 7.2 ± 0.2 × 10^−5^ min^−1^ μM^−1^) (Fig. 3*A*), and its specificity for its cognate substrate was reduced by the V697A substitution (*k*_cat_/K_M_ for RsbV-P increases 100-fold but was unaffected for SpoIIAA-P) (Fig. 3*A*).

SpoIIE^457–827^ exhibited several notable differences from the phosphatase domain alone (SpoIIE^590–827^). First, the regulatory domain increased the K_M_ of SpoIIE for both cognate and noncognate substrates, such that the concentration dependence of the phosphatase activity was linear even at concentrations of substrate above 100 μM (Fig. 3*A*). Second, the regulatory domain altered how the V697A substitution affected metal-cofactor binding; although the substitution decreased the concentration of MnCl_2_ required for SpoIIE^457–827^ to dephosphorylate SpoIIAA-P (Fig. 2*A*), it had no effect on the concentration of MnCl_2_ required to dephosphorylate RsbV-P (Fig. 3*B*).

Together, these findings (summarized in Fig. 3*C*) are consistent with coupling between substrate recognition, chemistry, and metal-cofactor recruitment and indicate that regulatory domains can influence all three steps through the switch.

### A role for the switch-element in activating RsbU phosphatase activity

**Figure 4:**
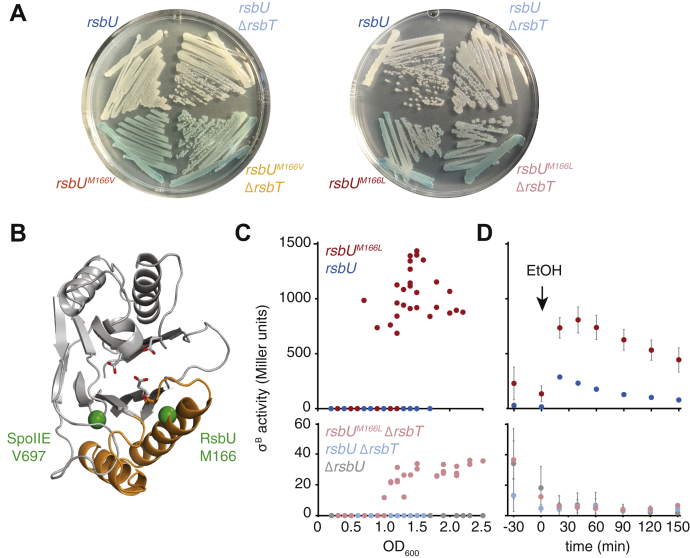
**Mutation of the switch activates RsbU activity in the absence of RsbT.***A*, RsbU mutants that are active in the absence of RsbT from a *blue*-*white* screen using a strain carrying a lacZ-reporter for σ^B^ activity. Colonies of cells expressing *rsbU*^*M166V*^ or *rsbU*^*M166L*^ at the native *rsbU* locus on the chromosome exhibit high levels of σ^B^ activity both in the presence and absence of *rsbT*. All strains lack *rsbPQ* and carry a reporter for σ^B^ activity (*amyE::ctc-lacZ*). Strains were grown on LB plates containing 80 μg/ml X-gal at 37 °C and imaged after 18 h. The presence of a *blue* pigment indicates σ^B^ activity and activation of the stress response pathway. *B*, a structure of the SpoIIE PP2C domain (PDB ID: 5UCG), with the cα of V697 and S640 (the equivalent of M166 in RsbU) displayed as *green spheres*. The α-helical switch is represented in *orange* and the side chains that coordinate divalent cations in the active site are shown as *sticks*. *C*, *rsbU*^*M166L*^ leads to increased σ^B^ activity as cultures approach the stationary phase. Plots show beta-galactosidase activity from *rsbPQ* deleted strains carrying a lacZ-reporter for σ^B^ activity (Δ*rsbPQ amyE::ctc*-lacZ) either encoding WT *rsbU* (*blue*), *rsbU*^*M166L*^ (*red*) (*top panel*), or deleted for *rsbU* (*gray*) (*bottom panel*). *Light colors* indicate that the strain additionally had *rsbT* deleted (*bottom panel*). The plots show cell density–dependent activation of σ^B^ from four independent replicates per strain background. Cultures were sampled from the early log phase to the stationary phase, and beta-galactosidase activity is plotted as a function of absorbance at 600 nm. *lacZ* expression was analyzed using ONPG as a substrate. Note: Y-axis scales differ between *top* and *bottom panels* to allow better visualization of data. *D*, *rsbU*^*M166L*^ leads to increased responsiveness to ethanol stress. Plots show beta-galactosidase activity from strains described in panels *C*. The plots show cell density–dependent activation of σ^B^ from four independent replicates per strain background. The complete data from each replicate can be viewed in Fig. S2B. Cultures were exposed to 4% ethanol (v/v) at low absorbance at 600 nm (∼0.1–0.2) to control for cell density–dependent effects on σ^B^ activity. Cultures were sampled at the early log phase 30 min and immediately before addition of ethanol and then at regular intervals afterward. *lacZ* activity analyzed as previously described and the mean beta-galactosidase activity is plotted as a function of time of ethanol addition. Error bars reflect SD from the mean. Y-axis scales differ between *top* and *bottom panels* to allow better visualization of data. LB, lysogeny broth; ONPG, *o*-Nitrophenyl-β-D-galactoside; PP2C, protein phosphatase 2C; X-gal, 5-bromo-4-chloro-3-indolyl-β-D-galactopyranoside.

Next, we addressed whether coupled activation, substrate recognition, and metal-cofactor binding through the switch is conserved for the stress-response phosphatase, RsbU (Fig. 1*A*). First, we tested the hypothesis that the switch mediates RsbT activation of RsbU. We took an unbiased approach to interrogate the mechanism of RsbU activation by performing a genetic screen to isolate variants of RsbU that are active in the absence of RsbT. We reasoned that if the switch is important for RsbU regulation, we would isolate activating mutations in the switch region. We generated a library of randomly mutagenized *rsbU* variants and induced their expression in a strain harboring a LacZ reporter for σ^B^ activity and selected for variants that activated σ^B^. From this screen, we identified two amino acid substitutions in the RsbU phosphatase domain, both at M166 (M166L and M166V), which activated σ^B^ in the absence of RsbT as visualized by *lacZ*-dependent color change on 5-bromo-4-chloro-3-indolyl-β-D-galactopyranoside (X-gal) plates (Fig. 4*A* shows plates with *rsbU*^*M116L/V*^ introduced at the native locus; Fig. S2*A* shows the plates with strains expressing *rsbU*^*M166L/V*^ using the plasmid-based system used in the screen). Activation of σ^B^ was dependent on expression of the mutagenized RsbU because both pathways known to dephosphorylate RsbV-P were deleted from the parental strain (and all other strains described below), and activation in plasmid-based systems depended on induction of RsbU expression (Fig. S2*A*). M166 is in the N-terminal α-helix of the switch and is predicted to pack in the hydrophobic core of the phosphatase domain (Fig. 4B). The position of these mutations is reminiscent of the activating mutation V697A in SpoIIE (Fig. 4*B*), and the position of M166 in the RsbU switch provides independent evidence that the switch is required for RsbU activation.

Our analysis of the SpoIIE V697A mutation revealed that it acts by biasing the switch toward the active conformation, allowing for partial activation in the absence of signal, and potentiating the response to low levels of signal (11). Consistent with a similar mechanism for the M166 mutations, we observed that when the plasmids used to isolate the *rsbU*^*M166L/V*^ variants were introduced to strains with *rsbT*, σ^B^ activity was elevated compared with *ΔrsbT* strains on plates (Fig. S2*A*). A similar phenomenon was not qualitatively apparent on plates for cells expressing *rsbU*^*M116L/V*^ at the native locus (Fig. 4*A*). However, we observed that the σ^B^ activity of liquid-grown cells expressing *rsbU*^*M116L*^ sharply increased between absorbance 0.5 and 1.0 at 600 nm and peaked as the cells entered the stationary phase (absorbance 1.5 at 600 nm) (Fig. 4*C*, top panel). This cell density–dependent effect was potentiated by RsbT; σ^B^ activity was 10-fold lower in strains that lacked *rsbT* (Fig. 4*C*, bottom panel). A similar cell density–dependent increase in σ^B^ activity was not detected in cells expressing WT *rsbU* regardless of the presence of *rsbT* (Fig. 4*C*, top panel). These data suggest that mutation of M166 sensitizes RsbU to activation by RsbT—even at levels that are normally insufficient to elicit a response.

The *rsbU*^*M166L*^ mutation also potentiated the RsbT-mediated response to environmental stress. Cells with *rsbU*^*M166L*^ integrated at the native chromosomal locus had an elevated response to a well-characterized environmental stressor (4% ethanol by volume) that activates RsbU through RsbT (12). Stress was introduced at low cell density (absorbance 0.1–0.2 at 600 nm) to minimize the impact of cell density on σ^B^ activity in *rsbU*^*M166L*^-expressing cells. The elevated response of cells expressing *rsbU*^*M166L*^ mirrored the characteristic dynamics of the sharp and transient increase in σ^B^ activity of WT cells followed by a gradual ramp-down of activities (Fig. 4*D* and Fig. S2*B*, top panel). As this response depends on both RsbT and RsbU, strains lacking either of these genes failed to respond to the stressor (Fig. 4*D*, bottom panel). Together, the position of M166 in the RsbU regulatory switch and its impact on RsbU activity implicate the switch as a conserved element to control phosphatase activity in response to cellular signals.

### Mechanisms of regulation and specificity are conserved between SpoIIE and RsbU

**Figure 5:**
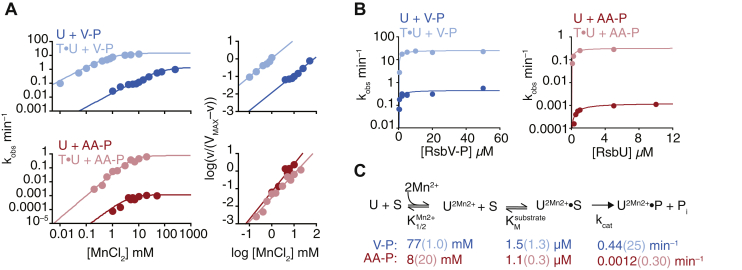
**RsbT modifies RsbU activity and specificity in a manner characteristic of switch-mediated activation.***A*, the plot of RsbU dephosphorylation of RsbV-P (*blue*, *top*) and SpoIIAA-P (*red, bottom*) in the presence (*light color*) and absence (*dark color*) of 10 μM RsbT. Reactions were performed at varying concentrations of MnCl_2_, and data are fit using nonlinear curve fitting (KaleidaGraph) to the equation k_obs_ = *k*_cat_∗[MnCl_2_]^*h*^/(K_1/2_ + [MnCl_2_]^*h*^). Values of *h* were determined from Hill plots of activity shown to the right (data were fit to a linear equation). For RsbU with RsbV-P, *h* is 1.0 (1.0 with T) and 1.4 with SpoIIAA-P (1.6 with T). For reactions with SpoIIAA as the substrate, single turnover reactions with 2.5-μM RsbU were performed (due to the slow rates of dephosphorylation). Reactions with RsbV-P as the substrate included 25-μM RsbU (single turnover in the absence of T) or 25 μM RsbV-P and 0.5 μM RsbU (multiple turnovers in the presence of T because of the rapid rate of dephosphorylation). *B*, plots of RsbU activity toward RsbV-P (*left*, *blue*) and SpoIIAA-P (*right*, *red*) in the absence (*dark colors*) and presence (*light colors*) of RsbT (10 μM). Curves are fits to the Michaelis–Menten equation rate = *k*_cat_∗[RsbU or RsbV-P]/(K_M_ + [RsbU or RsbV-P]). The *k*_cat_ for RsbV-P is 25 min^−1^ (0.4 min^−1^ without T) and K_M_ 1.3 μM (1.5 μM without T). The *k*_cat_ for SpoIIAA-P is 0.32 min^−1^ (0.0012 min^−1^ without T) and K_M_ 0.3 μM (1 μM without T). Multiple turnover reactions included 0.1-μM RsbU, and all reactions were performed with saturating MnCl_2_ (100 mM except for 10 mM for reactions of RsbV-P in the presence of T). *C*, a summary of the reaction scheme for RsbU dephosphorylation with values determined for K_1/2_ for MnCl_2_, K_M_ for substrate, and *k*_cat_ in the presence and absence of RsbT below. S indicates the phosphoprotein substrate, and P indicates the product.

If the switch element acts similarly to activate SpoIIE and RsbU, a simple prediction is that activation of RsbU by RsbT would decrease the concentration of the metal cofactor required for the activity. We reconstituted RsbU activation by RsbT and found that RsbT decreased the K_1/2_ of RsbU for Mn^2+^ by nearly 100-fold (K_1/2_ 1.0 ± 0.1 mM in the presence of RsbT and 77 ± 9 mM in the absence of RsbT) (Fig. 5*A*). In contrast to SpoIIE, RsbU activity toward its cognate substrate was noncooperative with respect to Mn^2+^ concentration (Fig. 5*A*, right panels). Although SpoIIE regulates a developmental transition, for which an all-or-nothing response is important, RsbU regulates a stress response, for which the graded response that noncooperative activation affords may be advantageous.

In our *in vitro* assays, RsbT additionally enhanced the maximal catalytic activity of RsbU toward RsbV-P 10-fold (*k*_cat_ 25 ± 2 min^−1^ in the presence of RsbT and 0.44 ± 0.06 min^−1^ in the absence of RsbT) (Fig. 5*A*), consistent with previous observations (12, 20). This suggests that, as we observed for SpoIIE, control of chemistry and metal-cofactor binding are coupled, providing nearly a 1000-fold increase in the activity of RsbU at concentrations of metal ion below the K_1/2_ (as would be expected in the cytoplasm) (21, 22).

Reconstitution of RsbU activation by RsbT further allowed us to determine how specificity is maintained upon phosphatase activation. Like SpoIIE, RsbU is specific: RsbU had a higher catalytic activity toward its cognate substrate (*k*_cat_ 0.44 ± 0.06 min^−1^ for RsbV-P and 0.0012 ± 0.0004 min^−1^ for SpoIIAA-P) and bound both cognate and noncognate substrates tightly (K_M_ 0.9 μM for SpoIIAA-P and 1.5 μM for RsbV-P; K_M_ values were difficult to determine accurately because of limitations of detection for substrate proteins) (Fig. 5*B*).

As with the cognate substrate, RsbT enhanced RsbU catalytic activity toward SpoIIAA-P (a 300-fold increase to *k*_cat_ 0.32 ± 0.02 min^−1^) (Fig. 5*B*). However, RsbT did not substantially change the affinity of metal-cofactor binding with the noncognate substrate (K_M_ 8 ± 3 mM in the absence of RsbT and 20 ± 6 mM in the presence of RsbT), similar to the V697A mutation of SpoIIE^457–827^ (Fig. 5*A*). We did note partial cooperativity for manganese with SpoIIAA as the substrate (Hill coefficient of 1.4 ± 0.1 without RsbT and 1.6 ± 0.3 with RsbT). This mild effect is consistent with our observation that the substrate influences the binding of metal in the active site. RsbT also had little impact on SpoIIAA-P binding (approximate K_M_ 1 μM in the absence of RsbT and 0.3 μM in the presence of RsbT) (Fig. 5*B*). Thus, specificity is maintained because RsbT activation fails to stimulate metal-cofactor binding for RsbU in the presence of its noncognate substrate (summarized in Fig. 5*C*).

## Conclusions

By examining two phosphatases, we found that PP2C regulation and specificity is mediated by a conserved allosteric element that is functionally flexible to respond to varied signals and to discriminate between substrate proteins. This regulatory mechanism is orchestrated by an allosteric switch that integrates activating signals and binding to cognate substrate to control metal-cofactor binding in the active site and catalytic activity. This coupling of regulatory inputs, chemistry, and substrate recognition provides a unified mechanism to control diverse signaling pathways.

By what mechanism could the PP2C switch element transduce regulatory inputs to coordinate substrate recognition, metal-cofactor binding, and chemistry? In a parsimonious model, both regulatory domains and substrates might directly contact and influence the conformation of the switch. This is supported by our findings that a fragment of the regulatory domain of SpoIIE shifts the energetic balance between substrate recognition, chemistry, and metal-cofactor recruitment. Furthermore, the switch is the structural feature of SpoIIE that mediates these changes in phosphatase activity and substrate recognition. The switch is situated directly below the active site, where it would likely contact substrate proteins and might also control the maximum catalytic rate by precise positioning of the phosphoserine bond and the metal cofactors in the active site. Such an architecture is present in the single available structure of a phosphatase from the PP2C family bound to its substrate protein (the phosphatase Hab1 bound to SnRK2). SnRK2 makes direct contacts with the Hab1 switch and to a variable region (termed “the flap”) that packs against the switch (23).

Dimerization may also be a widespread regulatory mechanism for the PP2C family of phosphatases. SpoIIE is activated by formation of a dimer in which the switch elements of the two phosphatase domains make direct contacts and lock the switch element in the active state (11). One attractive possibility is that the specific contacts between the switch elements in the phosphatase dimer are conserved. This is supported by the fact that similar contacts have been observed in dimeric structures of *Pseudomonas aeruginosa* phosphatases RssB and SiaA (24, 25). Dimerization also plays an important role in RsbU activation; a fragment of the RsbU regulatory domain crystallized as a dimer and a groove formed at the dimer interface was shown to be critical for RsbT binding to RsbU (20). However, the mechanism of how RsbU dimerization is coupled to phosphatase activation is unknown.

We hypothesize that this conserved allosteric switch may regulate diverse PP2C family of phosphatases across the tree of life. Regulatory domains from diverse PP2C family of phosphatases also pack against the switch, suggesting that coupling between substrate recognition, metal-cofactor binding, and activity could be broadly conserved (11, 26, 27, 28). In addition, structural analysis and molecular dynamics simulations of human PP2Cα revealed a network of interactions that link the switch element, metal binding in the active site, and the flap subdomain (29). Hydrogen–deuterium exchange experiments revealed increased mobility of the switch helices (α1 and α2) at subsaturating metal-cofactor concentrations and when the binding site for a third metal ion was disrupted by mutation of its coordinating residue, D146 (29). Further highlighting the similarities between the bacterial and human phosphatases, SpoIIE V697 and PP2Cα D146 both sit at the end of β-strand 6, two amino acids away from each other in a sequence alignment. These data suggest that the structural features of the PP2C catalytic domain that we identify as controlling phosphatase activity and specificity are broadly conserved.

The mechanism we describe here for PP2C regulation and specificity, however, is qualitatively different from what is known about evolutionarily unrelated serine/threonine phosphatases from other families. For members of the phosphoprotein phosphatase family including PP1, PP2A, and calcineurin, the catalytic domain itself is thought to be relatively promiscuous for substrates. Substrate specificity is instead imposed by diverse regulatory subunits that tether substrates in proximity of the catalytic center, control subcellular localization of the phosphatase, and/or gate substrate access to the active site (4).

Because phosphatases control signaling pathways that contribute to multiple disease processes, they are attractive drug targets, but their lack of deep and thus classically druggable active site pockets has made development of small-molecule modulators difficult (30). Recent successes, however, have targeted phosphatase regulatory mechanisms: PP1 inhibitors that target the conformation of regulatory subunits (31), PP2A activators that promote the assembly of specific regulatory subunits into holoenzymes (32), and allosteric modulators of active site gating for the protein tyrosine phosphatase, SHP2 (33). The allosteric regulatory mechanism of PP2C family of phosphatases may also similarly provide strategies for the development of modulators. Indeed, allosteric inhibitors of the oncogenic PP2C Wip1 were identified that bind to the flap subregion, although the mechanism of action of these inhibitors is unknown (34, 35). We propose that these and future PP2C modulators may act on the allosteric switch we here describe as coordinating substrate recognition, chemistry, and metal-cofactor binding.

## Experimental procedures

### Growth conditions

Liquid cultures were grown in Lennox lysogeny broth (LB), whereas colonies were grown on LB containing Bacto agar. The antibiotics used were macrolide-lincosamide-streptogramin (0.5 μg/ml erythromycin, 2.5 μg/ml lincomycin), chloramphenicol (20 μg/ml for *Escherichia coli*), carbenicillin (100 μg/ml), and kanamycin (10 μg/ml for *B. subtilis* or 50 μg/ml for *E. coli*). When indicated, 80 μg/ml of X-gal and 1 mM IPTG were added to the growth medium to visualize σ^B^ reporter activity on plates. *B. subtilis* strains are listed in Supplemental Table S1, *E. coli* strains are listed in Supplemental Table S2, and primers are listed in Supplemental Table S3.

### Protein-expression constructs

SpoIIAA and SpoIIAB were expressed in BL21 (DE3) Rosetta 2 pLysS cells as 6H-sumo fusions in pET23a made by ligation of the coding sequence in pET23a 6H-sumo digested with NotI/AgeI. SpoIIAA-P was produced in BL21 (DE3) cells containing pET-YSBLIC 6H-3C-spoIIAA–spoIIAB as reported (36). SpoIIE^590–827^, SpoIIE^457–827^, RsbT, RsbU, RsbV, and RsbW were produced with 3C cleavable 6H tags in BL21 (DE3) cells (constructs were made by insertion of the coding sequence to pET47b vectors digested with XmaI/XhoI by isothermal assembly). Mutations were introduced to SpoIIE expression constructs using the QuikChange site-directed mutagenesis kit. RsbV-P was produced by coexpressing RsbV and RsbW using a pET47b vector generated by isolation of the coding sequences for RsbV and RsbW from genomic DNA, yielding a construct with RsbV fused to a 3C cleavable 6H tag followed by untagged RsbW. All strains used are listed in the tables of strains (Supplemental Tables S1 and S2).

### Protein expression and purification

Proteins were expressed and purified using slight modifications to previous methods (11). All proteins were purified to greater than 95% purity as assessed by Coomassie Brilliant Blue–stained SDS-PAGE gel. Unless otherwise noted, protein expression was induced with 1 mM IPTG for 14 to 18 h at 14 °C in BL21 (DE3) cells that had been grown in LB to absorbance 0.6 at 60 at room temperature (RT). Details for purification of individual proteins are as follows:

*SpoIIE* (the same protocol for all variants) was purified from cell pellets lysed (using a microfluidizer) in 50 mM K•Hepes, pH 8.0, 200 mM NaCl, 10% glycerol (v/v), 0.5 mM DTT, 20 mM imidazole, and 0.1 mg/ml PMSF. Lysates were clarified by spinning for 30 min at 16,000 RPM in a Sorvall SS-34 rotor at 4 °C. Protein was bound to a HisTrap HP column on an AKTA FPLC, washed, and then eluted with a gradient to 200 mM imidazole. The 6H tag was cleaved overnight in dialysis with PreScission Protease, and the tag and protease were removed by flowing over Ni-NTA resin. SpoIIE was next purified on a Resource Q column equilibrated in 50 mM Hepes, pH 8.0, 100 mM NaCl, 2 mM EDTA, and 2 mM DTT and eluted with a gradient to 500 mM NaCl. The SpoIIE containing fractions were spin concentrated and gel-filtered on a 120 ml Superdex 75 column equilibrated in 20 mM K•Hepes, pH 8.0, 100 mM NaCl, 10% glycerol (v/v), and 2 mM DTT. Protein was spin-concentrated to approximately 400 μM and flash-frozen and stored at −80 °C.

*SpoIIAA* was expressed for 4 h at 37 °C. Cell pellets were resuspended in 20 mM K•Hepes, pH 7.5, 200 mM NaCl, 0.5 mM DTT, and 0.1 mg/ml PMSF (5 ml/l of culture). Cells were lysed using a cell disruptor in one-shot mode (Constant Systems), and lysates were clarified by spinning for 30 min at 16,000 RPM in a Sorvall SS-34 rotor at 4 °C. The protein was bound to Ni-NTA resin (1 ml/l of culture) on the column by gravity flow, washed with the buffer containing 20 mM imidazole, and eluted with 200 mM imidazole. The 6H-sumo tag was cleaved using 10 μl of a 100 μM stock of ULP1 in overnight dialysis at 16 °C to 20 mM K•Hepes, pH 7.5, 200 mM NaCl, 10% glycerol (v/v), and 0.5 mM DTT. The 6H-sumo tag was subtracted by flowing over Ni-NTA resin equilibrated in the cleavage and dialysis buffer. The cleaved protein was then spin-concentrated and gel-filtered on a 120 ml Superdex 75 column equilibrated in 50 mM K•Hepes, pH 8.0, 100 mM NaCl, 10% glycerol (v/v), and 2 mM DTT. The protein was concentrated to approximately 400 μM, flash-frozen, and stored at −80 °C.

*SpoIIAB* was expressed for 4 h at 37 °C. Cell pellets were resuspended in 50 mM K•Hepes, pH 7.5, 200 mM NaCl, 10 mM MgCl_2_, 0.5% Triton X-100 (v/v), 0.5 mM DTT, and 0.1 mg/ml PMSF (5 ml/l of culture). Cells were lysed using a cell disruptor in the one-shot mode (Constant Systems), and lysates were clarified by spinning for 30 min at 16,000 RPM in a Sorvall SS-34 rotor at 4 °C. The protein was bound to Ni-NTA resin (1 ml/l of culture) on the column by gravity flow, washed with the buffer containing 20 mM imidazole, and eluted with 200 mM imidazole. The 6H-sumo tag was left uncleaved to aid in removal from phosphorylation reactions. SpoIIAB was spin-concentrated and gel-filtered on a 120 ml Superdex 75 column equilibrated in 50 mM K•Hepes, pH 7.5, 175 mM NaCl, 10 mM MgCl_2_, 10% glycerol (v/v), and 2 mM DTT. The protein was concentrated to approximately 400 μM, supplemented to 50% glycerol (v/v), and flash-frozen and stored at −80 °C.

*SpoIIAA-P* was purified from cells coexpressing 6H-3C-SpoIIAA and untagged SpoIIAB for 4 h at 37 °C. Cell pellets were resuspended in 20 mM K•Hepes, pH 7.5, 200 mM NaCl, 0.5 mM DTT, and 0.1 mg/ml PMSF (5 ml/l of culture). Cells were lysed using a cell disruptor in the one-shot mode (Constant Systems), and lysates were clarified by spinning for 30 min at 16,000 RPM in a Sorvall SS-34 rotor at 4 °C. The protein was bound to a HisTrap HP column on an AKTA FPLC, was washed with the buffer containing 20 mM imidazole and 500 mM NaCl, and eluted with a gradient to 300 mM imidazole. The tag was cleaved using PreScission protease overnight dialysis at 4 °C. The cleaved protein was then spin-concentrated and gel-filtered on a 120 ml Superdex 75 column equilibrated in 50 mM K•Hepes, pH 8.0, 100 mM NaCl, 10% glycerol (v/v), 2 mM DTT. The protein was concentrated to approximately 400 μM, flash-frozen, and stored at −80 °C.

*SpoIIAB* was expressed for 4 h at 37 °C. Cell pellets were resuspended in 50 mM K•Hepes, pH 7.5, 200 mM NaCl, 10 mM MgCl_2_, 0.5% Triton X-100 (v/v), 0.5 mM DTT, and 0.1 mg/ml PMSF (5 ml/l of culture). Cells were lysed using a cell disruptor in the one-shot mode (Constant Systems), and lysates were clarified by spinning for 30 min at 16,000 RPM in a Sorvall SS-34 rotor at 4 °C. The protein was bound to Ni-NTA resin (1 ml/l of culture) on the column by gravity flow, washed with the buffer containing 20 mM imidazole, and eluted with 200 mM imidazole. The 6H-sumo tag was left uncleaved to aid in removal from phosphorylation reactions. SpoIIAB was spin-concentrated and gel-filtered on a 120 ml Superdex 75 column equilibrated in 50 mM K•Hepes, pH 7.5, 175 mM NaCl, 10 mM MgCl_2_, 10% glycerol (v/v), and 2 mM DTT. The protein was concentrated to approximately 400 μM, supplemented to 50% glycerol (v/v), and flash-frozen and stored at −80 °C.

*RsbT* was purified from cell pellets lysed (using a microfluidizer) in 20 mM K•Hepes, pH 7.5, 200 mM NaCl, 10% glycerol (v/v), 0.5 mM DTT, 20 mM imidazole, and 0.1 mg/ml PMSF. Lysates were clarified by spinning for 30 min at 16,000 RPM in a Sorvall SS-34 rotor at 4 °C. The protein was bound to Ni-NTA resin (1 ml/l of culture) on the column by gravity flow, washed, and eluted with 200 mM imidazole. The 6H tag was cleaved overnight in dialysis with PreScission protease, and the tag and protease were removed by flowing over Ni-NTA resin. RsbT was spin-concentrated and gel-filtered on a 120 ml Superdex 75 column equilibrated in 20 mM K•Hepes, pH 7.5, 150 mM NaCl, 10% glycerol (v/v), and 2 mM DTT. The protein was spin-concentrated to approximately 40 μM (additional concentration led to aggregation) and was flash-frozen and stored at −80 °C.

*RsbU* was purified from cell pellets lysed (using a microfluidizer) in 50 mM K•Hepes, pH 8.0, 200 mM NaCl, 10% glycerol (v/v), 0.5 mM DTT, 20 mM imidazole, and 0.1 mg/ml PMSF. Lysates were clarified by spinning for 30 min at 16,000 RPM in a Sorvall SS-34 rotor at 4 °C. The protein was bound to a HisTrap HP column on an AKTA FPLC, was washed, and then was eluted with a gradient to 200 mM imidazole. The 6H tag was cleaved overnight in dialysis with PreScission protease, and the tag and protease were removed by flowing over Ni-NTA resin. Cleaved RsbU was spin-concentrated and gel-filtered on a 120 ml Superdex 75 column equilibrated in 20 mM K•Hepes, pH 8.0, 100 mM NaCl, 10% glycerol (v/v), and 2 mM DTT. The RsbU-containing fractions were then purified on a RESOURCE Q column equilibrated in 50 mM Hepes pH 8.0, 100 mM NaCl, 2 mM EDTA, and 2 mM DTT and eluted with a gradient to 500 mM NaCl. This final step removes a degradation product present in some RsbU preparations. The protein was supplemented with 10% glycerol (v/v), spin-concentrated to approximately 400 μM, and flash-frozen and stored at −80 °C.

*RsbV* was purified from cell pellets lysed (using a microfluidizer) in 20 mM K•Hepes, pH 7.5, 200 mM NaCl, 10% glycerol (v/v), 0.5 mM DTT, 20 mM imidazole, and 0.1 mg/ml PMSF. Lysates were clarified by spinning for 30 min at 16,000 RPM in a Sorvall SS-34 rotor at 4 °C. The protein was bound to Ni-NTA resin (1 ml/l of culture) on the column by gravity flow, washed, and eluted with 200 mM imidazole. The 6H tag was cleaved overnight in dialysis with PreScission protease, and the tag and protease were removed by flowing over Ni-NTA resin. RsbT was spin-concentrated and gel-filtered on a 120 ml Superdex 75 column equilibrated in 20 mM K•Hepes, pH 7.5, 150 mM NaCl, 10% glycerol (v/v), and 2 mM DTT. The protein was spin-concentrated to approximately 400 μM and flash-frozen and stored at −80 °C.

*RsbW* was purified from cell pellets lysed (using a microfluidizer) in 20 mM K•Hepes, pH 7.5, 10 mM MgCl_2_, 200 mM NaCl, 10% glycerol (v/v), 0.5 mM DTT, 20 mM imidazole, and 0.1 mg/ml PMSF. Lysates were clarified by spinning for 30 min at 16,000 RPM in a Sorvall SS-34 rotor at 4 °C. The protein was bound to a HisTrap HP column on an AKTA FPLC, washed, and eluted with a gradient to 250 mM imidazole. The 6H tag was left uncleaved to aid removal after phosphorylation reactions. RsbW was then purified on a Resource Q column equilibrated in 50 mM Hepes, pH 7.5, 200 mM NaCl, 10 mM MgCl_2_, and 2 mM DTT and eluted with a gradient to 1 M NaCl. RsbW containing fractions were spin-concentrated and gel-filtered on a 120 ml Superdex 75 column equilibrated in 50 mM K•Hepes, pH 7.5, 150 mM NaCl, 10% glycerol (v/v), and 2 mM DTT. The protein was spin-concentrated to approximately 100 μM and flash-frozen and stored at −80 °C.

*RsbV-P* was purified from cells coexpressing 6H-3C-RsbV and untagged RsbW that were grown at 37 °C and induced with 1 mM IPTG for 4 h at 37 °C. Cells were lysed (using a microfluidizer) in 50 mM K•Hepes, pH 7.5, 50 mM KCl, 10% glycerol (v/v), 0.5 mM DTT, 20 mM imidazole, and 0.1 mg/ml PMSF. Lysates were clarified by spinning for 30 min at 16,000 RPM in a Sorvall SS-34 rotor at 4 °C. The protein was bound to a HisTrap HP column on an AKTA FPLC, washed, and eluted with a gradient to 250 mM imidazole. RsbVW-containing fractions were then gel-filtered using a 120 ml Superdex 75 column and fractions containing either RsbV or RsbVW complex were collected. The sample was supplemented with 5 mM ATP, 10 mM MgCl_2_, and PreScission protease and placed in dialysis in 200 ml of the buffer also supplemented with MgCl_2_ and ATP at RT overnight. The 6H tag and protease were removed by flowing over Ni-NTA resin and the protein was gel-filtered again on a Superdex 75 column equilibrated in 50 mM K•Hepes, pH 8.0, 100 mM NaCl, 2 mM DTT, and 10% glycerol (v/v). Free RsbV-P was collected, and complete phosphorylation was confirmed by isoelectric focusing. The protein was spin-concentrated to approximately 400 μM and flash-frozen and stored at −80 °C.

### Phosphatase assays

Phosphatase assays were performed using methods reported previously (9) with modifications as described. To produce ^32^P-labeled SpoIIAA-P, 75 μM SpoIIAA, 5 μM SpoIIAB, and 50 μCi of γ-^32^P ATP were incubated overnight at RT in 50 mM K•Hepes, pH 7.5, 50 mM KCl, 750 μM MgCl_2_, and 2 mM DTT. Unincorporated nucleotide was removed from the reaction mixture by buffer exchange using a Zeba spin column (Pierce) equilibrated in 20 mM K•Hepes, pH 7.5, 200 mM NaCl, and 2 mM DTT. The sample was then flowed over Q Sepharose resin to remove SpoIIAB. SpoIIAA-P was then buffer-exchanged to the buffer used for subsequent assays using a Zeba spin column equilibrated in 50 mM K•Hepes, pH 8.0, and 100 mM NaCl. Labeled SpoIIAA-P was aliquoted and frozen at −80 °C for future use.

To produce ^32^P-labeled RsbV-P, 50 μM RsbV, 5 μM RsbW, and 200 μCi of γ-^32^P ATP were incubated overnight at RT in 50 mM K•Hepes, pH 7.5, 50 mM KCl, 10 mM MgCl_2_, and 2 mM DTT. Unincorporated nucleotide was removed from the reaction mixture by buffer exchange using a Zeba spin column (Pierce) equilibrated in 50 mM K•Hepes, pH 8.0, 100 mM NaCl, 20 mM imidazole. 6H-tagged RsbW was then removed by binding to Ni-NTA resin. The flow-through fraction containing RsbV-P was then exchanged to the buffer used for subsequent assays using two successive Zeba spin columns equilibrated in 50 mM K•Hepes, pH 8.0, and 100 mM NaCl to remove all unincorporated nucleotide and free phosphate. Labeled RsbV-P was aliquoted and frozen at −80 °C for future use.

All phosphatase assays were performed at RT in 25 mM K•Hepes, pH 8, 100 mM NaCl, and 100 μg/ml BSA (to prevent protein sticking to tubes). The concentrations of enzyme, substrate, and MnCl_2_ were varied as indicated. 10 μM RsbT was additionally added to reactions as indicated. We note that the magnitude of effects reported for RsbT are lower limits because higher concentrations of RsbT could not be tested because of aggregation of RsbT at a high concentration. Reactions were stopped in 0.5 M EDTA, pH 8.0, and 2% Triton X-100 (v/v) and run on PEI-Cellulose TLC plates developed in 1 M LiCl_2_ and 0.8 M acetic acid, and imaged on a Typhoon (GE Life Sciences). Phosphatase assays were performed more than three independent times as separate experiments.

### Strain construction

*B. subtilis* strains were constructed using standard molecular genetic techniques (in the PY79 strain background) and were validated to contain the correct constructs by double-crossover recombination at the correct insertion site. All strains are used in this study are described in the table of strains. A σ^B^ reporter strain (KC479 (14)) was used as a parent strain for all *B. subtilis* strains. A parent strain (Δ*rsbPQ* Δ*rsbTU rsbV-FLAG amyE::ctc-lacZ*) was generated for the screen of *rsbT*-independent *rsbU* variants. To generate a marked deletion of *rsbPQ*, the genomic regions 500 bp upstream and downstream of *rsbPQ* were amplified and ligated to a kanamycin resistance cassette using standard SOE PCR techniques. The linear DNA fragment was inserted into KC479 using standard techniques. Transformants were selected based on kanamycin resistance and confirmed by colony PCR and sequencing. A clean deletion of *rsbTU* was generated by generating a gBlock (IDT DNA Technologies) of regions corresponding to 500 bp upstream of *rsbT* and 500 bp downstream of *rsbU* ligated together. This fragment was introduced into the HindIII and EcoRI sites of pminiMAD2 (37). Standard genetic techniques were used to excise the chromosomal copy of *rsbTU*. Mutants were confirmed by colony PCR and sequencing of the genomic region. Similarly, clean deletions of *sigB*, *rsbT*, and *rsbU* were generated, with the modification that we utilized a gBlock (IDT DNA Technologies) of regions corresponding to 500 bp upstream and 500 bp downstream of the appropriate genes. A single FLAG tag was introduced at the C-terminus of *rsbV* using the pminiMAD2 vector. Point mutations of the *rsbU* gene were similarly introduced using the pminiMAD2 vector and confirmed by colony PCR and sequencing.

An inducible expression vector (pKH001-Pspank-*rsbU*) was generated to screen for *rsbT*-independent variants of *rsbU*. A gBlock (IDT DNA Technologies) was generated containing a super-folding *gfp* gene flanked by AgeI and NotI sites and introduced into plasmid pDR110 (at the HindIII and SphI sites) using standard genetic techniques. The fragment of the resulting plasmid containing the *lacI* gene and the inducible *sfgfp* gene was PCR-amplified and inserted into vector pHB201 (38) at the SacI and KpnI sites using standard genetic techniques to generate pKH001-Pspank-*sfgfp*. To generate expression constructs for *rsbU* and *rsbTU*, the appropriate genes were PCR-amplified from genomic DNA from PY79 using a high-fidelity polymerase and introduced at the AgeI and NotI sites using isothermal assembly.

To generate libraries of pKH001-Pspank-*rsbU* containing mutagenized *rsbU*, the *rsbU* gene was PCR-amplified from genomic DNA from PY79 using error-prone PCR, utilizing the natural error rate of Taq polymerase. Mutagenized *rsbU* was introduced at the AgeI and NotI sites using standard genetic techniques. Plasmids were introduced into the parent strain (Δ*rsbPQ* Δ*rsbTU rsbV-FLAG amyE::ctc-lacZ*) using standard *B. subtilis* transformation techniques.

### Screen

Six independent pools of mutagenized *rsbU* were generated by amplifying *rsbU* under mutagenic PCR conditions using GoTaq polymerase (Promega) and an additional 2 mM MgCl_2_. These pools of mutagenized *rsbU* were subcloned into linearized plasmid using isothermal assembly to generate pKH001-P (spank)-*rsbU* and transformed separately into *E. coli* DH5α cells to propagate plasmids. The mutation rate was confirmed to be approximately one SNP per kilobase of DNA by sequencing selected clones. *E. coli* colonies were pooled to generate mixed populations of mutagenized pKH001-Pspank-*rsbU*, and this DNA was introduced separately into independent clones of the parent strain. Cultures were grown in selective media and plated on the selective medium containing IPTG and X-gal for 2 days at 37 °C. Approximately 12,000 mutant strains were analyzed. Blue colonies were selected, and phenotypes were confirmed by restreaking on an indicator medium before plasmids were miniprepped and sequenced. Mutation M166V was isolated independently in four of six pools of mutagenized *rsbU*, and M166L was isolated once. Mutations were reintroduced into pKH001-Pspank-*rsbU* through QuikChange PCR and introduced into fresh parent backgrounds to confirm that phenotypes were dependent on expression of mutated *rsbU*.

### Liquid-based beta-galactosidase assays

Beta-galactosidase assays to measure σ^B^ reporter activity were adapted from previously described protocols (39). In brief, cells were grown to desired absorbance, spun down, and frozen at −80 °C. Thawed pellets were resuspended in Z-buffer (60 mM Na_2_HPO_4_ 7•H_2_O, 40 mM NaH_2_PO_4_ H_2_O, 10 mM KCl, 1 mM MgSO_4_, 50 mM 2-mercaptoethanol) and kept on ice. Cells were arrayed in a 96-well plate, and the absorbance at 600 nm was measured to assess cell culture density. Cells were lysed in the Z-buffer containing a final concentration of 10 mg/ml lysozyme for 30 min at 37 °C. *o*-Nitrophenyl-β-D-galactoside was dissolved in Z-buffer (4 mg/ml) and added to lysed cells to a final concentration of 0.67 mg/ml. Absorbance at 420 nm (to measure *o*-nitrophenyl-β-D-galactoside cleavage) and 550 nm (to control for light scattering caused by cell debris) was measured over 40 min, and the rate of LacZ production was calculated from the slope of the linear phase of A_420_ − 1.75∗A_550_ (between 300 and 1500 s). Miller units were calculated using the formula that 1 Miller Unit = 1000∗ (A_420_ − (1.75∗A_550_))/(t∗v∗absorbance at 600 nm); t is time in minutes, and v is the volume of the reaction in milliliters.

### Figure preparation

Figures from structural models were generated using PyMol (Version 2.0) and UCSF Chimera (40). The sequence alignment in Fig. S1 was constructed and validated using UCSF Chimera and rendered using Geneious R7.

## Data availability

All data are contained within the article or available to be shared upon request (please contact the corresponding author).

## Supporting information

This article contains supporting information (1, 9, 11, 14, 36, 41).

## Conflict of interests

The authors declare that they have no conflicts of interest with the contents of this article.
