# Supplementary material for: A conserved allosteric element controls specificity and activity of functionally divergent PP2C phosphatases from Bacillus subtilis
Source: J Biol Chem. 2021 Mar 6;296:100518. doi: 10.1016/j.jbc.2021.100518 (PMC8080068; doi:10.1016/j.jbc.2021.100518)
Supplement: Supplemental Figures S1–S2 and Tables S1–S3 [file mmc1.pdf]

Supplementary Information for: A conserved allosteric element controls specificity and activity of functionally divergent PP2C phosphatases from *B. subtilis*

Kristin Ho[1] and Niels Bradshaw[1*]

[1]Department of Biochemistry, Brandeis University, Waltham, USA

* corresponding author

Supplementary materials included:

Supplementary Figure SF1

Supplementary Figure SF2

Supplementary Table S1

Supplementary Table S2

Supplementary Table S3

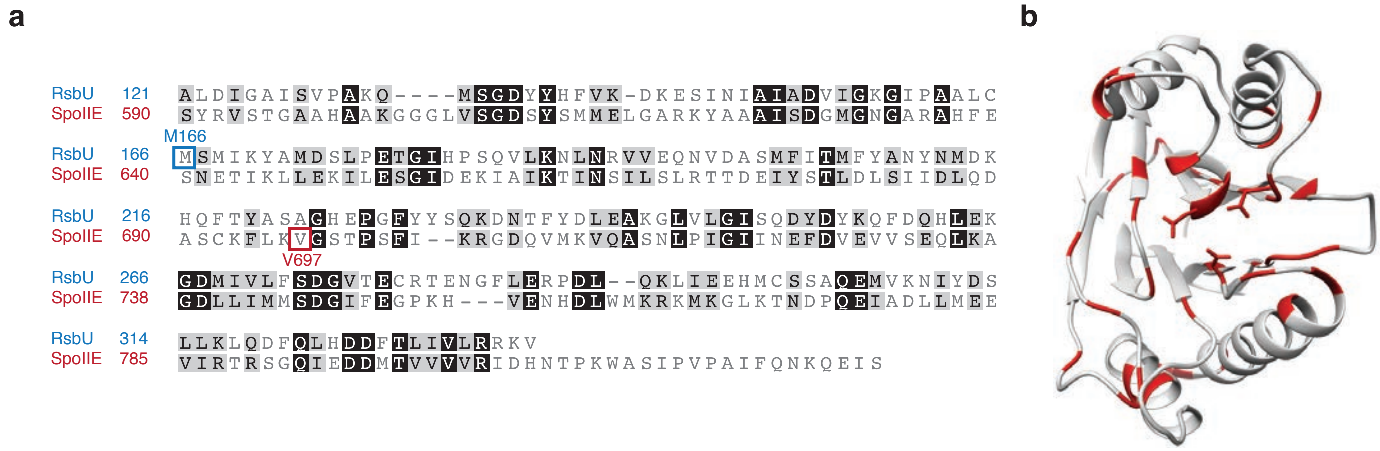

SF1. a. Alignments of the PP2C domains of RsbU and SpoIIE (generated using Geneious). Regions of identity are highlighted by black boxes and regions of similarity (according to Blosum62 scoring matrix) are highlighted by gray boxes. **b.** Crystal structure of the PP2C domain of SpoIIE (aa 590-827) (PDBID 5UCG). Regions of identity with the PP2C domain of RsbU are highlighted in red using the render by attribute tool in UCSF Chimera.

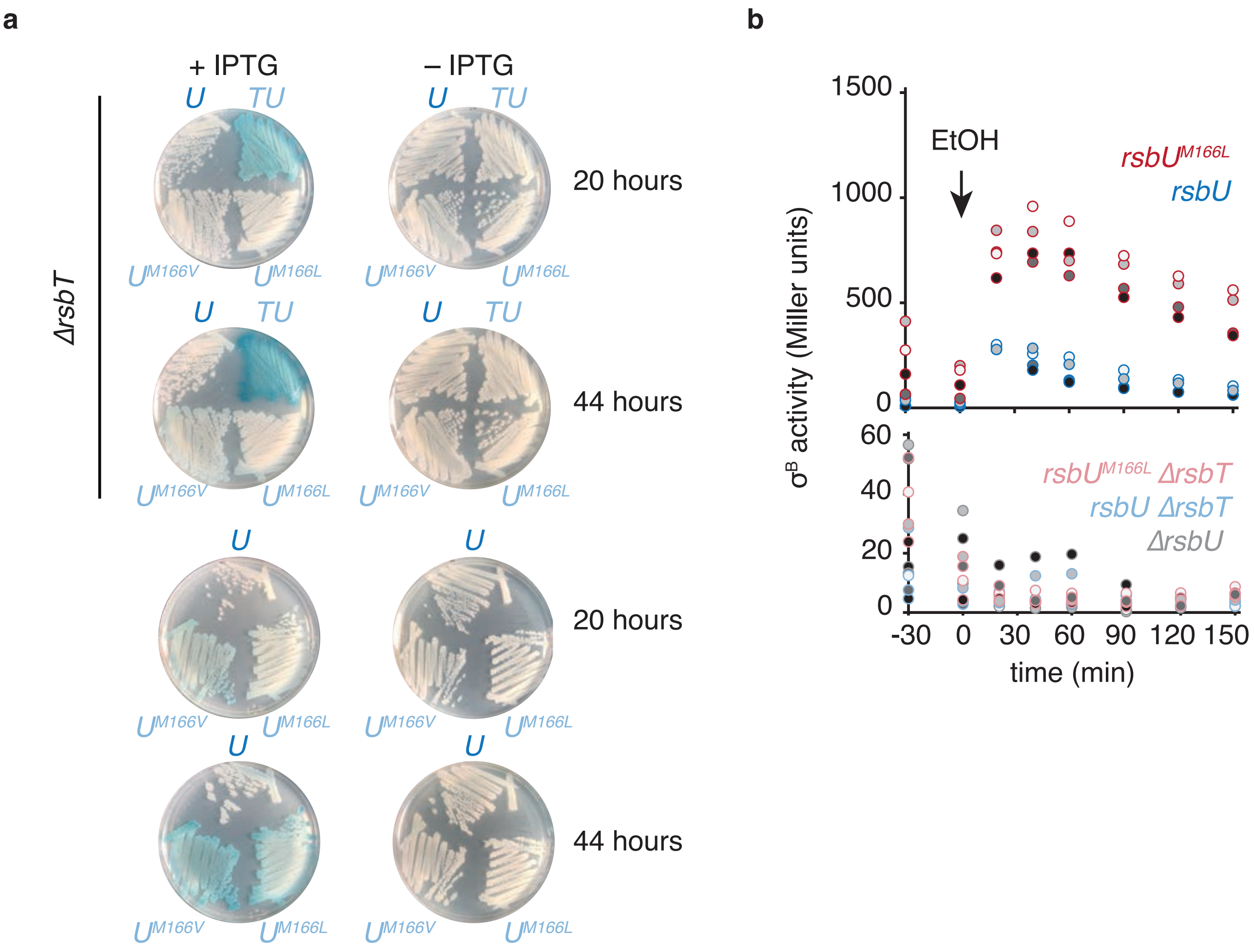

SF2. a. Plates validating the isolation of hyperactive RsbU variants from a blue-white screen for σ^B activity. All strains either lack *rsbT* (Δ*rsbPQ*Δ*rsbTU rsbV-flag amyE::ctc-lacZ*) (NB4) or encode native *rsbT* (Δ*rsbPQ*Δ*rsbU amyE::ctc-lacZ*) (NB10) and carry plasmids containing *rsbU* variants (wild-type *rsbU, , rsbU*^{M166V}, *rsbU*^{M166L}*)* (NB11, NB13, and NB14 (Δ*rsbT*) or NB15, NB16, and NB17 (+*rsbT*) respectively)*,* or both *rsbT* and *rsbU* under the control of an IPTG-inducible reporter (NB12). Strains were grown on LB plates containing MLS (for plasmid retention), 80 μg/mL X-gal, ±1 mM IPTG at 37ºC and imaged after 20 and 44 hours. Presence of blue pigment indicates σ^B activity and activation of the stress response pathway. **b.** These data are summarized in Figure 4D, and demonstrates that *rsbU*^{M166L} leads to increased responsiveness to ethanol stress. Plots show beta-galactosidase activity from *rsbPQ* deleted strains carrying a

lacZ-reporter for σ^B activity (Δ*rsbPQ amyE::ctc*-lacZ) either encoding wild-type *rsbU* (blue) (NB3), *rsbU*^{M166L} (red) (NB8) (top panel), or deleted for *rsbU* (NB10) (gray) (bottom panel). Light colors indicate that the strain additionally had *rsbT* deleted (NB9 for wild-type *rsbU* and NB6 for *rsbU*^{M166L} encoding strains) (botton panel). Cultures were exposed to 4% ethanol (v/v) at low OD_{600} (~0.1-0.2) to control for cell density-dependent effects on σ^B activity. Cultures were sampled at early log phase 30 minutes and immediately prior to addition of ethanol and then at regular intervals afterward. *lacZ* activity analyzed as previously described, and mean beta-galactosidase activity is plotted as a function of time of ethanol addition. Note: Y-axis scales differ between top and bottom panels to allow better visualization of data.

Supplementary Table S1:

B. subtilis **strains (all** ***B. subtilis*** **strains are in the background of PY79 (NB1))**

**Strain #**	**Genotype**	**Reference**
NB1	prototrophic wildtype (PY79)	(1)
NB2/KC479	*amyE::ctc-lacZ*	(14)
NB3	Δ*rsbPQ rsbV-FLAG amyE::ctc-lacZ*	This study
NB4	Δ*rsbPQ* Δ*rsbTU rsbV-FLAG amyE::ctc-lacZ*	This study
NB5	Δ*rsbPQ* Δ*rsbT* $rsbU^{M166V}$ *rsbV-FLAG amyE::ctc-lacZ*	This study
NB6	Δ*rsbPQ* Δ*rsbT* $rsbU^{M166L}$ *rsbV-FLAG amyE::ctc-lacZ*	This study
NB7	Δ*rsbPQ* $rsbU^{M166V}$ *rsbV-FLAG amyE::ctc-lacZ*	This study
NB8	Δ*rsbPQ* $rsbU^{M166L}$ *rsbV-FLAG amyE::ctc-lacZ*	This study
NB9	Δ*rsbPQ* Δ*rsbT rsbV-FLAG amyE::ctc-lacZ*	This study
NB10	Δ*rsbPQ* Δ*rsbU rsbV-FLAG amyE::ctc-lacZ*	This study
NB11	Δ*rsbPQ* Δ*rsbTU rsbV-FLAG amyE::ctc-lacZ pKH001 rsbU*	This study
NB12	Δ*rsbPQ* Δ*rsbTU rsbV-FLAG amyE::ctc-lacZ pKH001 rsbTU*	This study
NB13	Δ*rsbPQ* Δ*rsbTU rsbV-FLAG amyE::ctc-lacZ pKH001* $rsbU^{M166V}$	This study
NB14	Δ*rsbPQ* Δ*rsbTU rsbV-FLAG amyE::ctc-lacZ pKH001* $rsbU^{M166L}$	This study
NB15	Δ*rsbPQ* Δ*rsbU rsbV-FLAG amyE::ctc-lacZ pKH001 rsbU*	This study
NB16	Δ*rsbPQ* Δ*rsbU rsbV-FLAG amyE::ctc-lacZ pKH001* $rsbU^{M166V}$	This study
NB17	Δ*rsbPQ* Δ*rsbU rsbV-FLAG amyE::ctc-lacZ pKH001* $rsbU^{M166L}$	This study

Supplementary Table S2:

***E. coli* strains**

Strain	Genotype	Reference
NB18	*BL21 (DE3) Rosetta2 plysS pET23a 6H-sumo-spoIIAA*	(9)
NB19	*BL21 (DE3) Rosetta2 plysS pET23a 6H-sumo-spoIIAB*	(9)
NB20	*BL21 (DE3) pET-YSBLIC 6H-3C-spoIIAA spoIIAB*	(36)
NB21	*BL21 (DE3) pET47b 6H-3C-spoIIE590–827*	This study
NB22	*BL21 (DE3) pET47b 6H-3C-spoIIE590–827 V697A*	This study
NB23	*BL21 (DE3) pET47b 6H-3C-spoIIE457–827*	(11)
NB24	*BL21 (DE3) pET47b 6H-3C-spoIIE457–827 V697A*	(11)
NB25	*BL21 (DE3) pET47b 6H-3C-rsbT*	This study
NB26	*BL21 (DE3) pET47b 6H-3C-rsbU*	This study
NB27	*BL21 (DE3) pET47b 6H-3C-rsbV*	This study
NB28	*BL21 (DE3) pET47b 6H-3C-rsbW*	This study
NB29	*BL21 (DE3) pET47b 6H-3C-rsbVW*	This study
NB30	*DH5α pminiMAD2 ΔrsbTU*	This study
NB31	*DH5α pminiMAD2 ΔrsbU*	This study
NB32	*DH5α pminiMAD2 ΔrsbTU*	This study
NB33	*DH5α pminiMAD2 rsbV-FLAG*	This study
NB34	*DH5α pminiMAD2 ΔrsbT rsbUM166V*	This study
NB35	*DH5α pminiMAD2 ΔrsbT rsbUM166L*	This study
NB36	*DH5α pminiMAD2 rsbUM166V*	This study
NB37	*DH5α pminiMAD2 rsbUM166L*	This study
NB38	*DH5α pKH001 sfgfp*	This study
NB39	*DH5α pKH001 rsbU*	This study
NB40	*DH5α pKH001 rsbTU*	This study
NB41	*DH5α pKH001 rsbUM166V*	This study
NB42	*DH5α pKH001 rsbUM166L*	This study

Supplementary Table S3:

Primers used

Primer Sequence	Description
gaagtcctctttcagggacccggg**atgaacgaccaatcctgtgtaag**	forward primer to make *rsbT* in pET47b digested with XmaI/XhoI
gcagcctaggttaattaagcctcgag**ctaccgaagccatttgatgg**	reverse primer to make *rsbT* in pET47b digested with XmaI/XhoI
gaagtcctctttcagggacccggg**atggattttagggaggttattgagc**	forward primer to make *rsbU* in pET47b digested with XmaI/XhoI
gcagcctaggttaattaagcctcgag**ttaaacctttctccgcaaaacaat**	reverse primer to make *rsbU* in pET47b digested with XmaI/XhoI
gaagtcctctttcagggacccggg**atgaatataaatgttgatgtgaagcaaaac**	forward primer to make *rsbV* in pET47b digested with XmaI/XhoI
gcagcctaggttaattaagcctcgag**tcattgcactccaccttctg**	reverse primer to make *rsbV*v in pET47b digested with XmaI/XhoI
gaagtcctctttcagggacccggg**atgaagaataatgctgattacatcga**	forward primer to make *rsbW* in pET47b digested with XmaI/XhoI
gcagcctaggttaattaagcctcgag**ttagttagtttcgtagtttttgatggttg**	reverse primer to make *rsbW* in pET47b digested with XmaI/XhoI
gaaacagctatgaccatgattacgccaagcttgttgatcatcgggaacccggttaaatag	Forward primer for amplifying Δ*rsbPQ::knr* (upstream fragment)*;* HindIII site available
caattcgccctatagtgagtcgtgattggctcctttcagccggaaagtaatataa	Reverse primer for amplifying Δ*rsbPQ::Kn*^{R} (upstream fragment)*;* contains overhang for *Kn*^{R}
ccagcttttgttccctttagtgagaatgatccatgagacataacaatgatttgttacatc	Forward primer for amplifying Δ*rsbPQ::Kn*^{R} (downstream fragment); contains overhang for *Kn*^{R}
cacgacgttgtaaaacgacggccagtgaattcaatacgtacggcaaaaaagtcatggtgg	Reverse primer for amplifying Δ*rsbPQ::Kn*^{R} (downstream fragment); EcoRI site available
gaaacagctatgaccatgattacgcc	Forward primer for amplifying Δ*rsbPQ::Kn*^{R} cassette; HindIII site available
cacgacgttgtaaaacgacggc	Reverse primer for amplifying Δ*rsbPQ::Kn*^{R} cassette; EcoRI site available
gcgtatttaatcatggacacgcataaagccgctggaatg	QuikChange primer, rsbU bp A496G (M166V) mutation, forward
cattccagcggctttatgcgtgtccatgattaaatacgc	QuikChange primer, rsbU bp A496G (M166V) mutation, reverse

gcgtatttaatcatggacaagcataaagccgctggaatg	QuikChange primer, rsbU bp A496T (M166L) mutation, forward
cattccagcggctttatgcttgtccatgattaaatacgc	QuikChange primer, rsbU bp A496T (M166L) mutation, reverse
caattaagcttcatatcaaggaggaataccggtatgaacgaccaatcctgtgtaagaatc	Forward primer to make *rsbT* in pKH001 digested with AgeI/NotI
caattaagcttcatatcaaggaggaataccggtgtggattttagggaggttattgagcag	Forward primer to make *rsbU* in pKH001 digested with AgeI/NotI
ttccaccgaattagcttgcatgcgcggccgcttaaacctttctccgcaaaacaattaacg	Reverse primer to make *rsbU* in pKH001 digested with AgeI/NotI
